# Effects of moderate drought extension on bacterial network structure in the rhizosphere soil of *Leymus chinensis* in semi-arid grasslands

**DOI:** 10.3389/fmicb.2023.1217557

**Published:** 2023-08-10

**Authors:** Jinlong Wang, Chunjuan Wang, Xuefeng Wu, Jinwei Zhang, Guiyun Zhao, Yu Hou, Haiming Sun

**Affiliations:** ^1^College of Science, Traditional Chinese Medicine Biotechnology Innovation Center in Jilin Province, Beihua University, Jilin, China; ^2^Chongqing Institute of Quality and Standardization, Chongqing, China; ^3^Department of Grassland Science, College of Animal Science and Technology, Northeast Agricultural University, Harbin, China

**Keywords:** dry intervals, precipitation volume, soil bacterial community, precipitation gradient, semiarid grassland

## Abstract

**Introduction:**

Grasslands are home to complex bacterial communities whose dynamic interactions play a crucial role in organic matter and nutrient cycling. However, there is limited understanding regarding the impact of changes in rainfall amount and the duration of dry intervals on bacterial interactions.

**Methods:**

To assess the impact of changes in precipitation volume and dry intervals on bacterial co-occurrence networks, we carried out precipitation manipulation experiments in the Eastern Eurasian Steppe of China.

**Results and Discussion:**

We found that alterations in precipitation and dry intervals did not significantly affect bacterial alpha and beta diversity. However, we observed significant changes in the co-occurrence network structure of bacteria in the rhizosphere ecosystem, with the 12-day dry interval showing the most notable reduction in the number of degrees, edges, and clustering coefficient. Additionally, the study identified putative keystone taxa and observed that the moderately prolonged dry intervals between precipitation events had a major effect on the robustness of bacterial networks. The complexity and stability of the network were found to be positively correlated, and were primarily influenced by soil water content, phosphorous, and aboveground biomass, followed by available phosphorus (AP) and total biomass. These findings have the potential to enhance our comprehension of how bacterial co-occurrence pattern react to variations in dry intervals, by regulating their interactions in water-limited ecosystems. This, in turn, could aid in predicting the impact of precipitation regime alterations on ecosystem nutrient cycling, as well as the feedback between ecosystem processes and global climate change.

## 1. Introduction

Grasslands are among the most widespread terrestrial ecosystems, covering over 40% of the Earth’s land surface (Foley et al., 2005). Grassland ecosystems are vital components of global biogeochemical cycles and play an important role in supporting biodiversity and ecosystem services (Huguenin-Elie et al., 2019). These ecosystems are dominated by grasses and other herbaceous plants, which support a diverse range of wildlife and play a crucial role in global carbon and water cycles (Andersen et al., 2019). Bacterial communities are also important components of grassland ecosystems, playing key roles in nutrient cycling, soil structure, and plant-microbe interactions (Bardgett and Van Der Putten, 2014; Andersen et al., 2019). Changes in rainfall patterns, including alterations in the amount of rainfall and the length of dry intervals, can have significant effects on bacterial community structure in grassland soils (Zeglin et al., 2013; Chen et al., 2021). Therefore, understanding the impact of rainfall variability on bacterial communities is essential for predicting the response of grassland ecosystems to future climate change.

The effects of alterations in the amount of rainfall on bacterial community structure in grassland ecosystems are complex and multifaceted. Changes in rainfall patterns can affect the composition and diversity of bacterial communities, as well as their functional roles in ecosystem processes. Reduced rainfall can lead to a decrease in bacterial diversity and a shift in community structure, as seen in studies by De Vries et al. (2012) and Bell et al. (2013). On the other hand, increased rainfall can lead to changes in bacterial community composition and diversity, as well as an increase in nutrient availability, which can stimulate bacterial growth and metabolism (Ducousso-Détrez et al., 2022). However, the effects of rainfall alterations on bacterial community structure are not consistent across different grassland ecosystems, and can vary depending on the specific environmental conditions of each ecosystem. The effects of rainfall alterations on bacterial community structure in grassland ecosystems can exhibit variability across different sites, as demonstrated by several studies. For instance, a study by Zhang et al. (2016) investigated the responses of microbial communities to altered precipitation in the Tibetan plateau alpine grassland and found that bacterial community composition was positively related to soil moisture. Similarly, Bell et al. (2009) explored the responses of soil bacterial communities to altered soil moisture patterns in a Chihuahuan Desert Grassland, revealing significant changes in the composition of soil bacteria throughout different seasons. In addition, the effects of rainfall alterations on bacterial communities can interact with other environmental factors, such as nutrient availability, soil pH, and temperature, which can further complicate the relationships between rainfall and bacterial community structure (Wang et al., 2022). These studies provide evidence for the variable responses of bacterial communities to rainfall alterations across different grassland sites. However, most studies have focused on bacterial community composition and diversity, the understanding of interactions among microbial assemblages, microbiome complexity, and stability in response to different rainfall amounts and dry interval lengths remains limited. Further investigations are needed to unravel these intricate relationships.

Dry intervals, or periods of low soil moisture, are a common occurrence in many grassland ecosystems, particularly those in arid and semi-arid regions. These dry intervals can have significant effects on the microbial communities in soil, as they can limit nutrient availability and alter the physical structure of the soil. Bacterial communities are particularly sensitive to changes in soil moisture, and changes in their composition and diversity can have significant implications for ecosystem functioning. Studies have shown that the length of dry intervals can have significant effects on the composition and diversity of bacterial communities in grassland soil. For example, a study by Canarini et al. (2021) found that prolonged drought periods led to changes in the relative abundance of certain bacterial taxa in a grassland ecosystem, which in turn affected the availability of certain nutrients in the soil. Similarly, a study by Wang S. et al. (2021) found that a longer dry interval led to a decrease in the diversity of bacterial communities in a grassland ecosystem, which was associated with a decrease in soil organic carbon content.

The establishment of a microbial community is influenced by the interplay of various community assembly processes, as explained by Stegen et al. (2015). Selection, in particular, is a crucial ecological mechanism that drives the formation of microbial community structures. Interactions between microbial species, acting as a selective force, also contribute to the assembly of microbial communities (Hunt and Ward, 2015) and affect biogeochemical cycling (Morriën et al., 2017). Recent studies have revealed an increasing number of microorganism network structures in different environments (Wu et al., 2021; Yun et al., 2021; Zhou et al., 2021), which can shed light on the complexity of microbial assemblages. By utilizing microbial network analysis, researchers have been able to gain new insights into the structure of microbial communities (Wang et al., 2018) beyond traditional alpha and beta diversity metrics. Ecological network properties, which reflect the interactions among coexisting organisms, can impact community responses to environmental variations, including climate extremes (Wagg et al., 2019). Network analysis is an useful methodological tool for in-depth microbial community ecology analysis (Shi et al., 2016), as it provides additional information beyond simple abundance richness and composition metrics. However, there is still limited understanding of the interactions between members of microbial assemblages, microbiome complexity, and stability in various precipitation gradients. Therefore, it is valuable to comprehend how bacterial co-occurrence patterns respond to variations in precipitation. This understanding is necessary for predicting the effects of changes in precipitation regimes on the organization and dynamics of microbial interactions and niches, as well as biogeochemical cycling.

Despite the growing body of research on the effects of rainfall alterations on bacterial communities in grassland ecosystems, there are still many knowledge gaps and uncertainties that need to be addressed. To better understand the effects of the amount of rainfall and the length of dry intervals on microbial interactions and community assembly in semiarid grasslands, we conducted a precipitation manipulation experiment in a semiarid grassland in northern China. We used Gephi 0.9.2 and R to construct integrated co-occurrence networks of bacterial community datasets from 60 samples associated with different amount of rainfall and the length of dry intervals. In this paper, we investigate the effects of the length of dry intervals on bacterial community structure in a grassland ecosystem. Specifically, we ask the following questions: (1) How does the amount of rainfall and the length of dry intervals affect the co-occurrence patterns of bacterial communities in grassland soil? (2) Are these effects dependent on other environmental factors, such as soil pH and soil nutrient? (3) What are the potential implications of changes in bacterial community structure for nutrient cycling and ecosystem services in grassland ecosystems?

## 2. Methods

### 2.1. Study site and plant material

The present study was conducted in the central region of the Songnen grassland, specifically at the Songnen Grassland Ecological Research Station of Northeast Normal University in Changling County, Jilin Province, China (44°45′N, 123°45′E). This area is an experimental land designated by Northeast Normal University for the purpose of grassland science research. The Eastern Eurasian Steppe region experiences a typical mesothermal monsoon climate characterized by cold and dry winters and relatively warm and wet summers (Zhong et al., 2017; Shi et al., 2019). The annual mean temperature ranges from 4.6°C to 6.4°C, while annual precipitation ranges from 280 to 400 mm, with approximately 80% of precipitation events occurring from June to August in the last five decades (1961–2010). The mono-dominated species in this area is Leymus chinensis (Trin.) Tzvel. (Herbarium of Northwest A&F University (WUK, 0442655)). *L. chinensis* is a perennial clonal plant that primarily relies on vegetative propagation for population renewal and is highly palatable to livestock such as cattle and sheep (Gao et al., 2020). Thus, understanding the impact of rainfall variation on *L. chinensis* growth is crucial for predicting its production in this region.

### 2.2. Experiment design and field manipulation

To manipulate the amounts of precipitation and dry intervals between precipitation events from June to August 2018, we employed a two-factor randomized complete block design. Based on historical rainfall data obtained from the Changling County Meteorological Bureau, we used three levels of controlled precipitation. The long-term average biologically effective precipitation during the period from June 1st to September 1st was 334 mm (R0). We also used a 30% decrease (R-) and a 30% increase (R+) relative to the long-term average biologically effective rainfall, resulting in rainfall levels of 233 mm and 434 mm, respectively (Heisler-White et al., 2008). Biologically effective events were defined as those with daily precipitation equal to or greater than 2 mm, and cases of more than three consecutive days of precipitation were divided into two events (Heisler-White et al., 2008). At this site, the dry intervals between rainfall events ranged from 8.6 days to 13.3 days. As the IPCC predicts prolonged dry intervals between rainfall events in the future (Stocker et al., 2014), we selected five levels of dry intervals for this study: 6 days, 9 days, 12 days, 15 days and 18 days.

To accurately determine the amounts and intervals of rainfall, we conducted a simulated rainfall experiment using an arched rainout shelter with steel frames and clear polyethylene roofs. We removed the litter and dug out 24 cm diameter and 25 cm depth plant–soil cores from the selected patch. For this study, we had 18 treatments (3 × 5) with four replicates each, totaling 60 (15 × 4) plant–soil cores. Each plant–soil core was carefully transferred to a plastic pot (24 cm diameter and 26 cm height), which was sufficient for plant root growth since the rooting system of *L. chinensis* is mainly within 0–10 cm soil depth. Before the experiment, plants were allowed to acclimatize to their pots for 15 days and were watered every 3 days (a total of five times) to ensure survival and even growth (about 11.4 ± 1.3 cm high for plants in each pot). Soil water content values, measured with a TRIME Pico64 (IMKO. GmbH. Ettlingen. Germany), were similar in each pot (6.03 ± 0.52% v v − 1). The experimental water volume was measured using a measuring cylinder, and we used a watering can to simulate natural precipitation. Watering occurred between 6:30 and 9:30 AM. The shelter’s roof was exclusively employed during precipitation occurrences, and therefore, as soon as the weather cleared, we promptly proceeded with the removal of the polyethylene roof.

### 2.3. Soil sampling

We measured the soil volume water content in each pot using a TRIME Pico64 (IMKO.GmbH. Ettlingen. Germany) field moisture TDR-sensor every one to 2 days between 16:00–17:00 pm at a depth of 10 cm. The mean soil water content (SWC) was calculated by averaging the values obtained during the experiment. At the end of the treatment, we carefully removed the plant material from each pot and took three soil cores (diameter 2 cm, depth 25 cm) which were mixed in sealed bags to create one composite sample. The soil samples were transported to the laboratory in a cooler, homogenized and sieved through 2 mm mesh. After removal of roots, one part of the sample was kept at −20°C for measuring soil ammonium nitrogen content (NH4 + -N) and nitrate nitrogen content (NO3 − -N), while the other part was air-dried for 15 days and used to measure available P concentration. The concentrations of soil NH4 + -N and NO3 − -N were analyzed using a continuous flow analyzer (Alliance Flow Analyzer, Futura, Frépillon, France), while the available P was determined by the molybdate blue colorimetric method following extraction with 0.5 mol L − 1 NaHCO3.

### 2.4. DNA extraction and polymerase chain reaction

We utilized the FastDNA^®^ Spin Kit for Soil (MP Biomedicals, United States) to extract genomic DNA from 0.5 g of soil. PCR amplification of the V4 region of bacterial 16S was then performed with the primer pair 338F (5′-ACT CCT ACG GGA GGC AGC A-3′) and 806R (5′-GGA CTA CHV GGG TWT CTA AT-3′). The amplicons from different sites were pooled at equimolar concentrations after quantification. Sequencing was conducted using Illumina MiSeq, following the standard protocols of Majorbio BioPharm Technology Co., Ltd. (Shanghai, China). The processed sequences underwent operational taxonomic unit (OTU) assignment, employing the UPARSE software, based on a 97% sequence similarity threshold. Taxonomic assignments were conducted for each OTU using the ribosomal database project (RDP) Classifier algorithm(Cole et al., 2009), aligned against the Silval32 16S rRNA database, with a confidence threshold set at 70%.

The raw fastq files underwent demultiplexing and quality filtering using Trimmomatic. Subsequently, the files were merged using FLASH (Magoč and Salzberg, 2011), based on the following criteria: (1) Reads were truncated if their average quality score over a 50-bp sliding window fell below Q20. (2) Sequences containing more than one ambiguous character or two nucleotide mismatches in the primers were excluded. (3) Sequences with an overlap length of at least 10 bp were merged based on their overlap sequence Operational taxonomic units (OTUs) were clustered using UPARSE (Edgar, 2013) at 97% similarity (version 7.1, available at http://drive5.com/uparse/). Chimeric sequences were identified and eliminated using UCHIME. To determine the taxonomy of each gene sequence, the Ribosomal Database Project (RDP) Classifier algorithm^1^ (Cole et al., 2009) was employed, with a confidence threshold of 70%. Further information on DNA extraction, quality control, and raw data processing can be found in Wang et al. (2015).

### 2.5. Statistical analysis

A two-way analysis of variance (ANOVA), followed by Tukey’s test, was utilized to examine the influence of rainfall amounts and intervals, as well as their interaction, on the microbial Shannon index and subnetwork properties. To evaluate the impact of rainfall amounts and intervals, along with their interaction, on the microbial community structure, nonmetric multidimensional scaling (NMDS) and PERMANOVA (Martinez Arbizu, 2020) (‘adonis’ function, vegan package, version 2.6–4 in R) (Oksanen et al., 2019) were performed.

Co-occurrence network analysis was employed to investigate the interactions among soil bacterial species across varying dry intervals. The analysis focused on the operational taxonomic units (OTUs) level to construct co-occurrence networks of bacterial communities. To reduce the complexity of the data sets, only OTUs with more than 20 reads were retained for network construction. Only robust (*r* > 0.8 or *r* < −0.8) and significant Spearman correlations (*p* < 0.01) calculated within the “picante” R package (Xue et al., 2018) were incorporated into the network analyses (Hu et al., 2017). We adjust all *p*-values for multiple testing using the false discovery rate (FDR) according to Benjamini controlling procedure (Benjamini et al., 2006). Gephi^2^ version 0.9.3 with a Fruchterman-Reingold layout was used to visualization the co-occurrence networks across five levels of dry intervals. Networks are randomly colored by modules, and the size of the nodes is proportional to the degree. Sub-networks were created for each sample from sub-network by preserving OTUs in each sample with subgraph functions in the igraph package (version 1.4.2) (Wu et al., 2021), to identify the variance of topological features in bacteria networks. The network nodes were divided into four sub-categories based on within-module connectivity (Zi) and among-module connectivity (Pi): (1) peripherals (Zi < 2.5, Pi <0.62), (2) network hubs (Zi > 2.5, Pi >0.62), (3) module hubs (Zi > 2.5, Pi <0.62), and (4) connectors (Zi < 2.5, Pi >0.62) (Olesen et al., 2007). Network natural connectivity was evaluated by removing nodes in the static network to estimate the rate of robustness degradation, and network robustness was assessed based on the natural connectivity of the nodes, as it provides a sensitive discrimination for measuring network stability (Peng and Wu, 2016). Finally, random forest analysis was performed to determine the relative importance of physicochemical parameters in driving bacterial network structures. Random Forest analyses exhibit robustness against overfitting and insensitivity towards outliers (Leach et al., 2018). They possess the capability to handle datasets containing a large number of potential predictors. One significant advantage of Random Forest analyses is their ability to detect non-linear relationships without the requirement of prior specification (Jones and Linder, 2015). Furthermore, Random Forest analyses excel in accurately identifying influential predictors of the response even when there is high collinearity among predictors (Archer and Kimes, 2008). Additionally, they provide accurate predictions of the response variable (Cutler et al., 2007). The ‘importance’ function was used to measure the significance of factors by the value of % IncMSE (increased mean squared error). The above analysis and plotting were conducted using the random forest function of randomForest (version 4.7–1.1) package of R (Wang et al., 2023).

## 3. Results

### 3.1. Overview of community structure

Following quality control, we successfully obtained a total of 1,761,264 sequences from 60 samples via Illumina MiSeq sequencing (ranging from 31,871 to 69,689 reads per sample). We identified a total of 2,395 operational taxonomic units (OTUs), which were classified into 31 phyla, 78 classes, 184 orders, 275 families, 490 genera, and 949 species. The *Actinobacteriota* (36.03%), *Acidobacteriota* (15.57%), *Proteobacteria* (15.30%), *Chloroflexi* (12.91%), *Firmicutes* (5.06%), and *Gemmatimonadota* (4.95%) were the most abundant phyla detected. Additionally, our research findings revealed that variations in rainfall amounts and dry intervals did not have a significant impact on bacterial alpha diversity (Supplementary Figure S1 and Supplementary Table S1). However, we observed significant effects of dry intervals on beta diversity, with a gradual decline in these metrics observed from 6 days to 12 days, followed by a subsequent gradual increase from 12 days to 18 days (Supplementary Figure S2). The species accumulation curves (Supplementary Figure S3) tended to reach saturation plateaus as the sample number increased, indicating that the number of bacterial sequences obtained represented the bacterial communities well.

**Figure 1:**
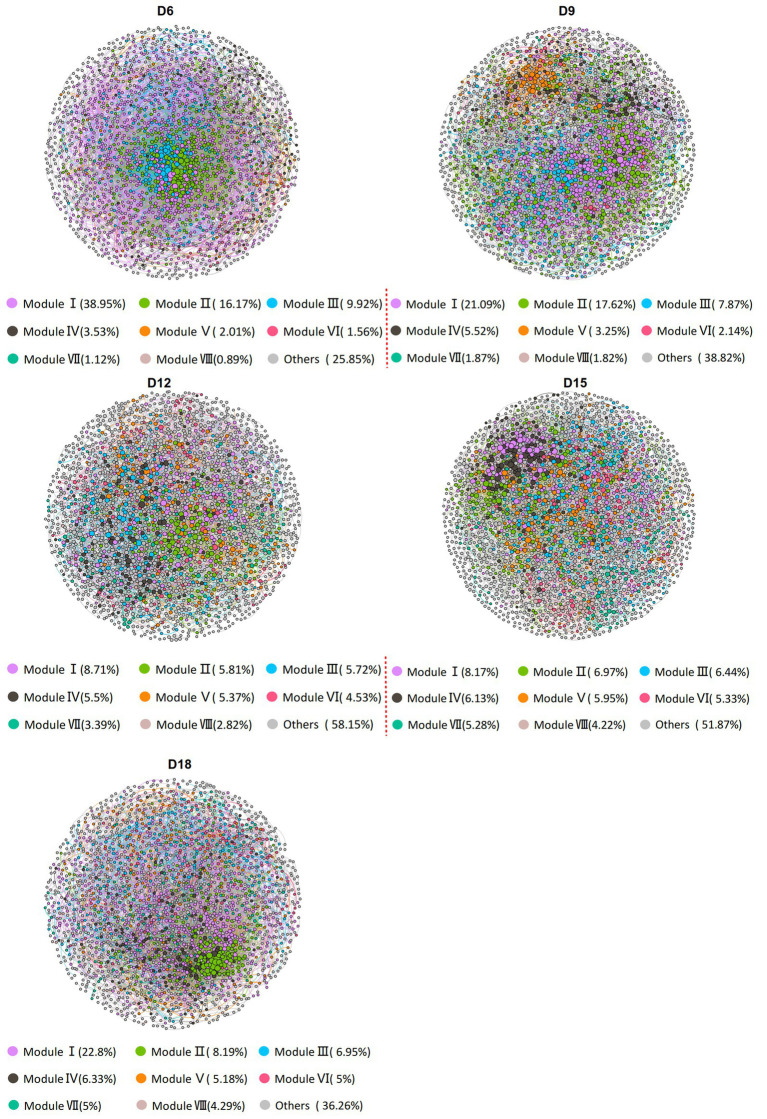
Co-occurrence networks of bacterial communities in rhizosphere soil of *Leymus chinensis* at different length of the dry intervals. Networks are randomly colored by modules, and the size of the nodes is proportional to the degree. D6: 6 days dry intervals; D9: 9 days dry intervals; D12: 12 days dry intervals; D15: 15 days dry intervals; D18: 18 days dry intervals.

### 3.2. Co-occurrence network structures

**Figure 2:**
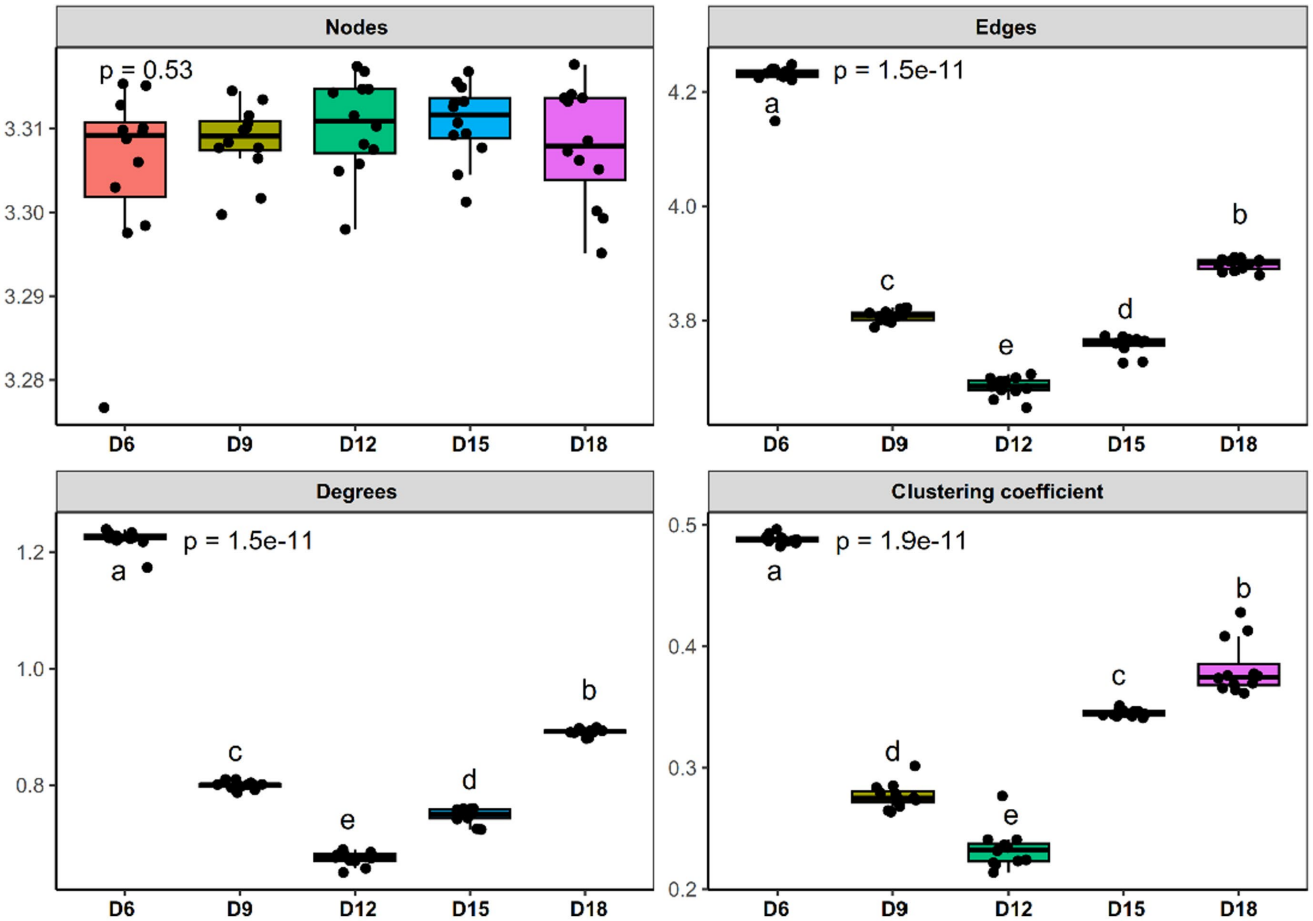
Comparison of node-level topological features of bacterial community co-occurrence patterns in rhizosphere soil of *Leymus chinensis* among five different length of the dry intervals. Significant differences (*p* < 0.05) between different EOR application oil reservoirs are marked with different letters. D6: 6 days dry intervals; D9: 9 days dry intervals; D12: 12 days dry intervals; D15: 15 days dry intervals; D18: 18 days dry intervals. Different lowercase letters indicate significant differences among the three sites (*p* < 0.05).

To evaluate the impact of the amount of rainfall and the length of the dry intervals on the co-occurrence network structure of bacteria, we computed a range of network-level topological properties to describe the complexity of the co-occurrence network. We found that there was no interaction effect between rainfall amount and dry interval on the bacterial sub-network topological parameters of *L. chinensis*, and only the dry interval has a significant impact on the bacterial network structure (Supplementary Table S2). Thus, five bacterial networks for the 6 days, 9 days, 12 days, 15 days and 18 days were constructed individually (Figure 1). After conducting our analysis, we discovered that the sub-networks in 12 days exhibited a notable reduction in the number of degrees, edges, and clustering coefficient, compared to other dry intervals. Furthermore, we observed a gradual decrease in these metrics from 6 days to 12 days, followed by a gradual increase from 12 days to 18 days (Figure 2 and Supplementary Table S2). These results demonstrate that bacterial co-occurrence patterns differed significantly among the length of dry intervals (*p* < 0.05). The interaction pattern of bacteria in 12 days dry treatment was less compact and aggregated than that in other dry intervals.

### 3.3. Module hubs and connectors as putative keystone taxa

**Figure 3:**
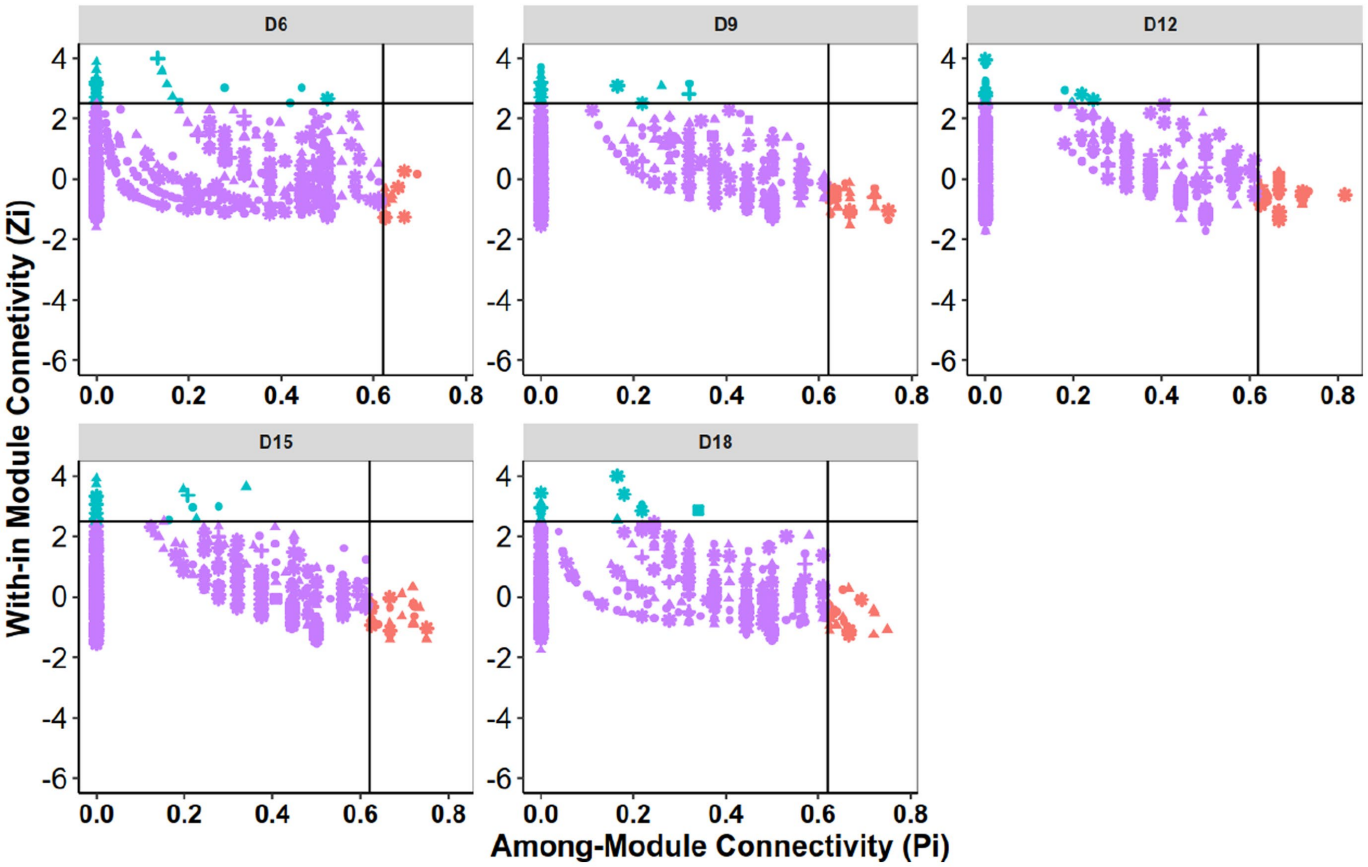
Zi-Pi plots to identify putative keystone OTUs within the bacterial community networks. Each point represents an OTU. Module hubs are identified as Zi ≥ 2.5, Pi <0.62; connectors are identified as Zi < 2.5, Pi ≥0.62. D6: 6 days dry intervals; D9: 9 days dry intervals; D12: 12 days dry intervals; D15: 15 days dry intervals; D18: 18 days dry intervals.

The roles of individual nodes in the network were determined using their within-module connectivity (Zi) and among-module connectivity (Pi) values. Each node was classified as either a peripheral, connector, module hub, or network hub. However, in this study, no network hubs were detected (Figure 3). A significant proportion of nodes were classified as peripherals, indicating that the majority of nodes in the network had few connections and were mostly linked only to nodes within their own modules. On average, peripherals accounted for over 94.8% of the total nodes in the five bacterial networks, while connectors and module hubs accounted for 3.4 and 2.0%, respectively. Notably, 78, 129, 137, 124, and 112 nodes were identified as keystone (connector and module hub) taxa in D6, D9, D12, D15, and D18, respectively. When dry intervals were prolonged, the number of keystone taxa initially increased but then decreased (Supplementary Table S3).

Module hubs and connectors have been proposed to be keystone taxa due to their important roles in network topology (Deng et al., 2012). Based on this criterion, members of *Acidobacteriota*, *Actinobacteriota* and *Proteobacteria* phyla would be the most prominent keystone taxa in the rhizosphere networks, as they accounted for approximately 28.5, 171% and 14.35 of all network hubs and connectors. In addition, putative keystone taxa include taxa from the phyla *Firmicutes, Chloroflexi, Bacteroidota, Gemmatimonadota* (Supplementary Table S3).

### 3.4. Effect of precipitation on natural connectivity of bacterial communities

**Figure 4:**
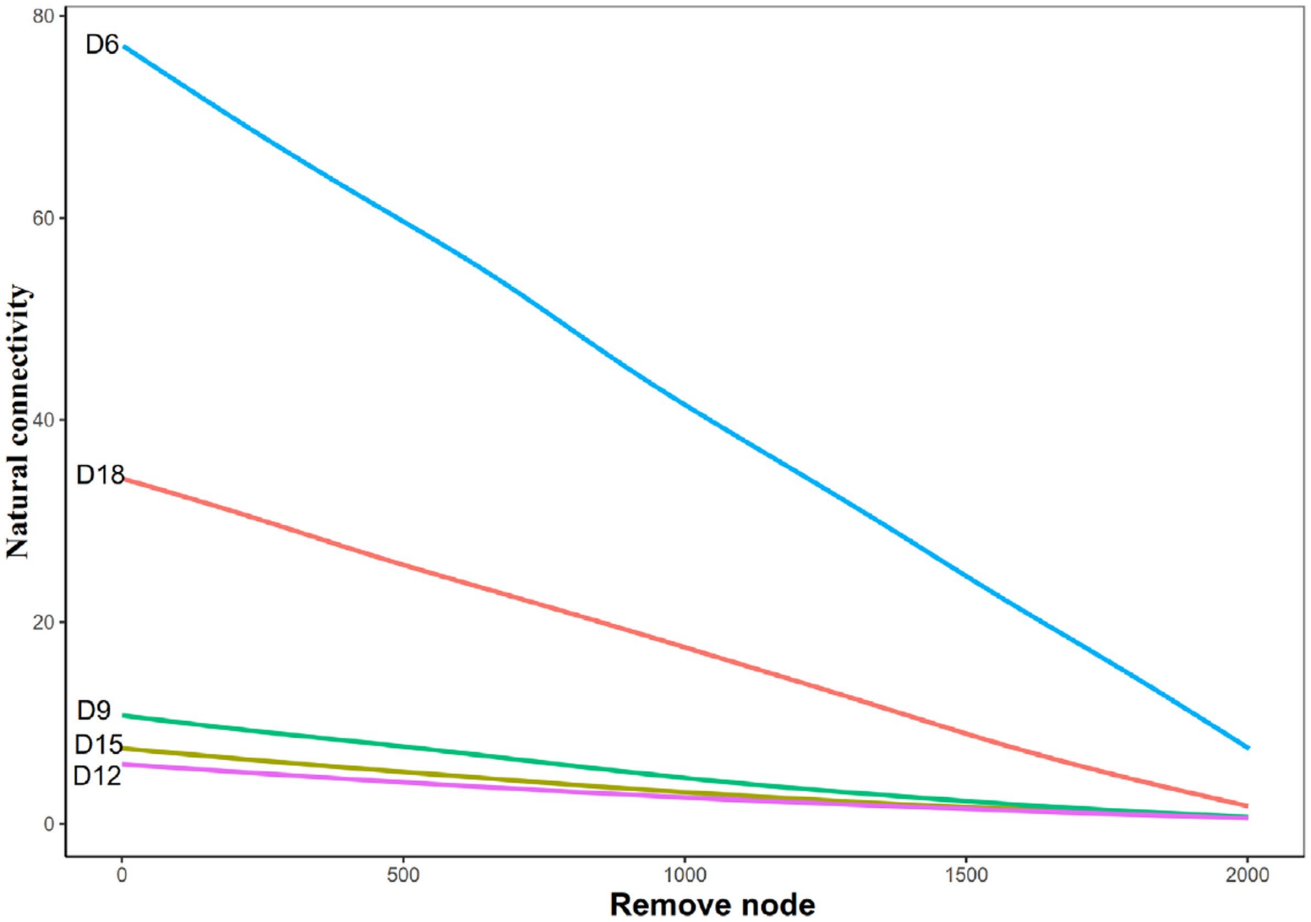
Natural connectivity of bacterial networks in rhizosphere soil of *Leymus chinensis* at different length of the dry intervals. D6: 6 days dry intervals; D9: 9 days dry intervals; D12: 12 days dry intervals; D15: 15 days dry intervals; D18: 18 days dry intervals.

Natural connectivity analysis was used to test the robustness of the network of bacteria in different days of dry intervals. The results show that for bacterial communities, networks in D6 intervals had the highest value of natural connectivity, followed by the networks in D18, D9, D15 and D12 (Figure 4). We also observed a gradual decrease in these metrics from D6 to D12, followed by a gradual increase from d12 to d18 (Figure 2). These results indicate that moderately prolonged dry intervals (D12) between precipitation events have a major effect on the robustness of bacterial networks. The stability of the network shows a consistent trend with the complexity (represent by degrees and links) of the network, a gradual decrease from 6 days to 12 days, followed by a gradual increase from 12 days to 18 days.

### 3.5. Linking network-level topological features to soil and plant properties

The study showed that alterations in the amount of rainfall, the length of dry intervals, and their interaction significantly affected the soil water content (SWC). Increased precipitation levels led to significant increases in SWC (Supplementary Table S4). However, when dry intervals were prolonged, SWC initially increased but then decreased. The rainfall amount and dry interval length had significant main effects on the available soil NH_4_^+^-N, and NO_3_^−^-N. Elevated rainfall levels resulted in decreased available soil NH_4_^+^-N, NO_3_^−^-N, and P. However, with prolonged dry intervals, the trend was a decrease followed by an increase (Supplementary Table S4).

**Figure 5:**
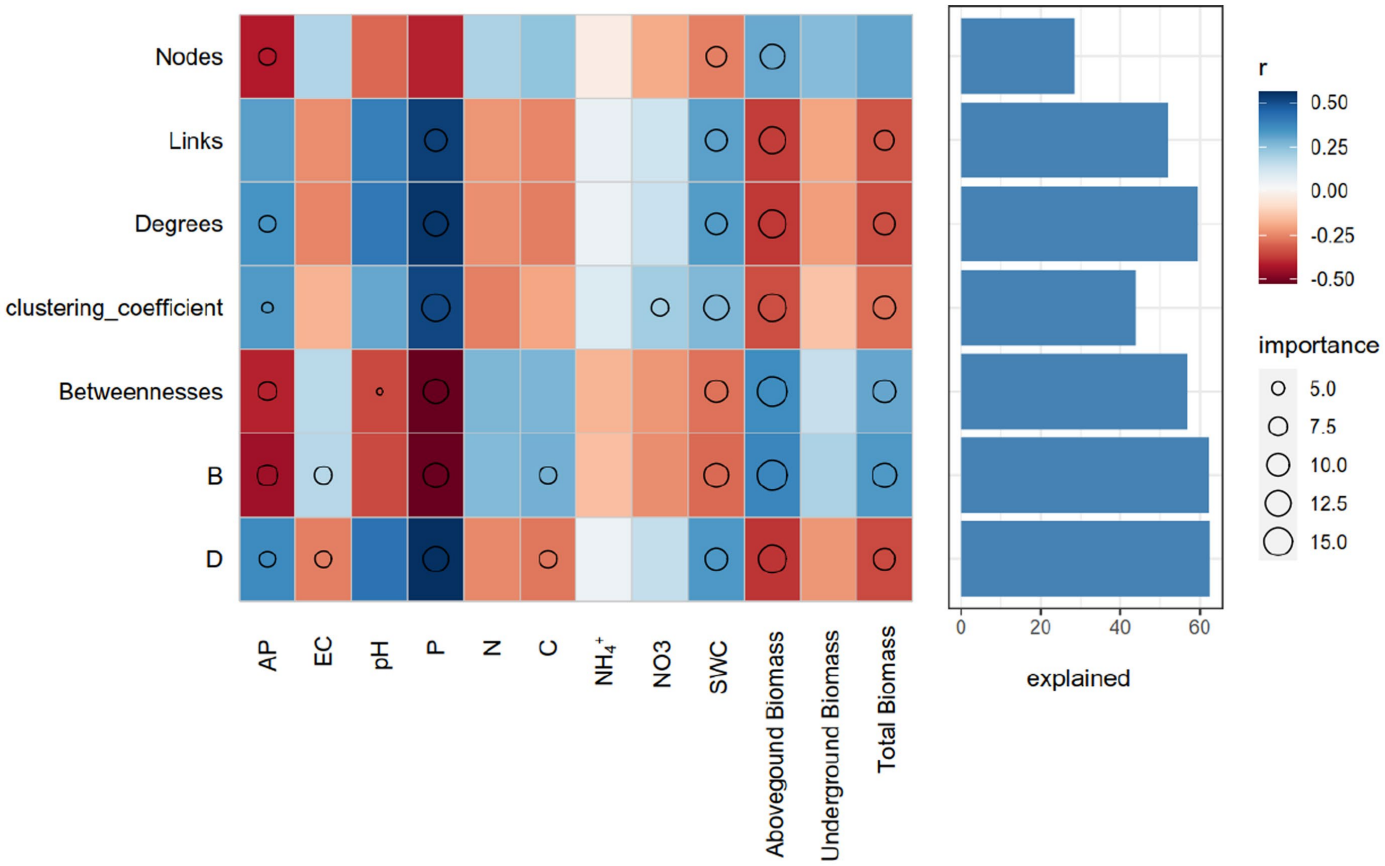
Potential contributions of the soil properties and plant aboveground and belowground biomass to the dissimilarities of network topological features. Circle (*p* < 0.05) size represents variable importance (i.e., the proportion of explained variation calculated via multiple regression modeling and variance decomposition analysis). Colors represent Spearman correlations. EC, elecstrical conductivity; C, soil total carbon; N, soil total nitrogen; P, soil total phosphorus; SWC, soil moisture.

We further evaluated the relative contribution of soil physicochemical parameters and plant properties to the topological features of networks for different length of dry intervals. Random forest analysis was performed to identify correlations between the network topological features and soil and plant factors. SWC, P and aboveground biomass were the main factors influencing the topological features of the bacterial networks. AP and total biomass were also important environmental attributes controlling the soil bacterial network structure (Figure 5). Link number, average degree, and clustering coefficient were positively correlated with P and SWC, belowground biomass and total biomass were negatively correlated with betweenness (Figure 5).

## 4. Discussion

In this study, we investigated the impact of changes in rainfall amount and the length of dry intervals on the co-occurrence network structure of bacterial communities in a rhizosphere environment. Our analysis revealed that the dry interval had a significant impact on the bacterial network structure, while the rainfall amount did not have a significant effect. Microorganisms exhibit high sensitivity to fluctuations in soil water availability and physicochemical conditions, particularly in semiarid grassland ecosystems (Chen et al., 2019; Yang et al., 2021). In water-limited environments like arid and semiarid grasslands, water availability stands as a crucial factor influencing soil microbial activity and nutrient (Wang et al., 2018). Precipitation variations can directly impact soil bacterial communities by altering soil water availability and indirectly by affecting soil nutrient availability, plant community composition, and productivity (Wu et al., 2020). However, our investigation revealed no discernible disparities in bacterial communities between increased and decreased precipitation events. This lack of differentiation can likely be attributed to the fact that both increased and decreased precipitation within short time frames resulted in alterations to the composition of bacterial communities by affecting the relative abundance of different bacterial species, which exhibited sensitivity to precipitation. This finding aligns with prior research that has emphasized the substantial influence of soil moisture content on the structure and diversity of microbial communities (Wang J. et al., 2021). Previous studies have indicated that changes in precipitation can directly influence soil bacterial communities through modifications to soil water availability (Bagayoko et al., 2000), primarily due to the sensitivity of certain taxa to fluctuations in water availability (de Vries et al., 2018). The shifts in bacterial interactions may arise from variations in the capacity of different species to adapt to changes in soil water availability.

We observed that the bacterial co-occurrence patterns differed significantly among D6, D9, D12, D15 and D18 networks. The findings suggest that dry intervals have a significant impact on the co-occurrence network structure of bacteria, and the length of the dry interval is more important than the amount of rainfall. The sub-networks in the D12 treatment exhibited a notable reduction in the number of edges, and clustering coefficient, compared to other dry intervals. This result suggests that moderately prolonged dry intervals (D12) between precipitation events have a significant effect on the network complexity and stability of bacterial communities. This is congruent with previous findings studies that have shown that drought conditions can lead to changes in the microbial community structure and co-occurrence patterns (Cruz-Martínez et al., 2012; Fuchslueger et al., 2014). Furthermore, These findings are consistent with earlier studies that have shown that moderate changes in soil moisture content can have a more significant impact on microbial communities than extreme changes (Bell et al., 2013; Geyer et al., 2019). The decrease in network complexity observed in the sub-networks during the 12-day dry interval may suggest that the microbial community is more delicate and vulnerable to environmental fluctuations. It is possible that soil microbes are working to establish stable networks capable of adapting to prolonged periods of drought. The gradual decline in these metrics from 6 to 12 days, followed by a subsequent increase from 12 to 18 days, suggests that the 12-day mark may represent a crucial period during which microbes work to reconstruct their network structure.

The use of network analysis in ecological studies has greatly enhanced our understanding of the structure and dynamics of microbial communities. One important aspect of network analysis is identifying key nodes in the network that are critical for maintaining network stability and functioning. In this study, the roles of individual nodes in the bacterial co-occurrence network were determined using their within-module connectivity (Zi) and among-module connectivity (Pi) values. The nodes were classified as either a peripheral, connector, module hub, or network hub. However, network hubs were not detected in this study, and a significant proportion of nodes were classified as peripherals. The high percentage of peripheral nodes in the networks indicates that most nodes in the network had few connections and were mostly linked only to nodes within their own modules. This finding is conforming to prior research results that have shown that most nodes in ecological networks are peripheral (Olesen et al., 2007; He et al., 2017; Wang et al., 2018). On average, peripherals accounted for over 94.8% of the total nodes in the five bacterial networks, while connectors and module hubs accounted for 3.4 and 2.0%, respectively. These findings imply that the majority of the nodes in the bacterial networks possibly played a non-essential role in maintaining network stability and functionality. However, keystone taxa, which are defined as taxa with a disproportionate influence on network structure and functioning (Deng et al., 2012), were identified in this study. A total of 78, 129, 137, 124, and 112 nodes were identified as keystone (connector and module hub) taxa in D6, D9, D12, D15, and D18, respectively. Interestingly, when dry intervals were prolonged, the number of keystone taxa initially increased but then decreased. This suggests that keystone taxa may play a more important role in maintaining network stability and functioning during short dry intervals, while their importance may decrease as dry intervals become longer. The phyla *Acidobacteriota*, *Actinobacterio*ta, and *Proteobacteria* were identified as the most prominent keystone taxa in the rhizosphere networks, accounting for approximately 28.5, 17, and 14.35% of all network hubs and connectors. These phyla are known to be common in soil and have been previously identified as keystone taxa in microbial networks (Barberán et al., 2012). Putative keystone taxa from the phyla *Firmicutes*, *Chloroflexi*, *Bacteroidota*, and *Gemmatimonadota* were also identified in the study.

The analysis of natural connectivity provides important insights into the robustness and stability of the bacterial networks in response to different length of dry intervals. The results suggest that the bacterial network in the rhizosphere soil of *Leymus chinensis* during D6 had the highest natural connectivity, which indicates that the network was highly connected and robust to perturbations. However, networks during D12 had the lowest natural connectivity, suggesting that the network was less connected and more vulnerable to perturbations. This finding is matching with previous investigations, which have demonstrated that the stability of ecological networks is closely related to their connectivity (Montoya et al., 2006; Thébault and Fontaine, 2010).The gradual decrease in natural connectivity from D6 to D12 followed by a gradual increase from D12 to D18 also supports the hypothesis that moderately prolonged dry intervals have a significant impact on the stability of bacterial networks. This result is consistent with the previous studies that have shown that the frequency and intensity of environmental disturbances can significantly affect the structure and stability of ecological networks (Fontaine et al., 2003; Thébault and Fontaine, 2010). Additionally, the decrease in natural connectivity observed in this study suggests that there might be a threshold of dry interval length beyond which the bacterial network becomes highly vulnerable to perturbations. This threshold might differ depending on the specific ecosystem and bacterial community being studied. Overall, the results of the natural connectivity analysis suggest that the stability and robustness of bacterial networks are highly dependent on the length of dry intervals. This finding is consistent with the broader literature on ecological networks, which has shown that network stability is a function of the frequency and intensity of environmental disturbances (Dunne et al., 2002; Thébault and Fontaine, 2010). Understanding the factors that affect the stability of ecological networks is critical for predicting the response of ecosystems to environmental change and designing effective management strategies for maintaining ecosystem functioning and biodiversity.

The use of machine learning techniques such as random forest analysis to identify the relative contribution of environmental and plant factors to the topological features of bacterial networks is a valuable approach in ecological research. The findings from this study suggest that soil water content, phosphorus availability, and plant properties such as aboveground biomass and total biomass are important factors influencing the topological features of bacterial networks. These results are in agreement with previous studies that have shown the strong influence of soil physicochemical parameters on microbial community structure and diversity (Lauber et al., 2009; Fierer et al., 2012). Additionally, a study investigated the impact of altered precipitation patterns on microbial interactions in semi-arid grassland soils in Northern China, revealing that mean annual precipitation was the primary factor influencing the network structure at a regional scale, while soil conditions and plant parameters played a more significant role at a local scale, with the desert network exhibiting a simpler structure and weaker association with environmental factors, indicating that microbial communities in extremely dry ecosystems are unstable and susceptible to future climate change (Wang et al., 2018). Soil water content is known to be a major factor affecting microbial activity and diversity, as it affects nutrient availability and oxygen diffusion in the soil (Bardgett and Van Der Putten, 2014). Similarly, plant properties such as aboveground biomass and total biomass are known to influence the microbial community structure and activity, through root exudation and nutrient cycling (Koranda et al., 2023). The positive correlation between link number, average degree, and clustering coefficient with P and SWC observed in this study suggests that bacterial networks are more complex and interconnected in soils with higher water content and phosphorus availability. This finding is consistent with previous studies that have shown the positive correlation between soil nutrient availability and microbial network complexity (Zhou et al., 2011; Shi et al., 2015). The negative correlation between belowground biomass and betweenness observed in this study suggests that bacterial networks are more centralized in soils with lower root biomass. This finding is in agreement with previous studies that have shown the negative correlation between plant root biomass and microbial network complexity (Banerjee et al., 2018).

In conclusion, this study demonstrated that bacterial community networks exhibited significant variations in their topological features across different dry intervals. Specifically, the interaction pattern of bacteria during the 12-day dry treatment was more compact and aggregated compared to other dry intervals, indicating that moderately prolonged dry intervals have a considerable impact on the robustness of bacterial networks. The complexity and stability of the network were found to be positively correlated, and were primarily influenced by SWC, P, and aboveground biomass, followed by AP and total biomass. Furthermore, our results showed that bacterial network structures became more stable but less complex in response to the 12-day dry treatment, which could potentially impact the ecosystem functions of grassland soils. Overall, these findings enhance our understanding of how changes in precipitation affect bacterial interactions in semiarid grassland ecosystems, providing valuable insights for predicting the effects of precipitation changes on ecosystem nutrient cycling and the feedback between ecosystem processes and global climate change.

## Data availability statement

The datasets presented in this study can be found in online repositories. The names of the repository/repositories and accession number(s) can be found below: NCBI - PRJNA988419.

## Author contributions

The overall project was proposed and organized by JW, CW, and XW. JZ and CW conducted the majority of experiments, with assistance from JW, GZ, and YH in laboratory work and analyses. The primary text of this manuscript was authored by JW and HS, while discussions were contributed to by XW, CW, JZ, and YH Interpretation of results and final manuscript review and editing were carried out by JW, HS, and XW. All authors contributed to the article and approved the submitted version.

## Funding

This work was supported by Northeast Agricultural University Young Talents Program (22QC11), Jilin Province Science and Technology Development Program Project (20230101189JC), and PhD research start-up project of Beihua University.

## Conflict of interest

The authors declare that the research was conducted in the absence of any commercial or financial relationships that could be construed as a potential conflict of interest.

## Publisher’s note

All claims expressed in this article are solely those of the authors and do not necessarily represent those of their affiliated organizations, or those of the publisher, the editors and the reviewers. Any product that may be evaluated in this article, or claim that may be made by its manufacturer, is not guaranteed or endorsed by the publisher.

## Footnotes

^1^ http://rdp.cme.msu.edu/

^2^ https://gephi.org/
