# Supplementary material for: Effects of moderate drought extension on bacterial network structure in the rhizosphere soil of Leymus chinensis in semi-arid grasslands
Source: Front Microbiol. 2023 Aug 10;14:1217557. doi: 10.3389/fmicb.2023.1217557 (PMC10448527; doi:10.3389/fmicb.2023.1217557)
Supplement: Supplementary file 2 [file Data_Sheet_1.docx]

**Moderately Extension of Drought Interval in Semi-arid Grasslands Promotes Stability and Reduces Complexity of Bacterial Network Structure in Leymus chinensis.**

Jinlong Wang^a,1^, Chunjuan Wang^a,1^, Xuefeng Wu^c^, Jinwei Zhang^b,^*, Guiyun Zhao^a,*^ , Yu Hou^a^, Haiming Sun^a^

^a^ College of Science, Traditional Chinese Medicine Biotechnology Innovation Center in Jilin Province, Beihua University, Jilin 132013, China

^b^ Chongqing Institute of Quality & Standardization, Chongqing 400000, China

^c^ Department of Grassland Science, College of Animal Science and Technology, Northeast Agricultural University, Harbin, China

^1^These authors have contributed equally to this work
*Correspondence: Jinwei Zhang, zhangjw133@nenu.edu.cn; Guiyun Zhao, 476112258@qq.com

**Table S1.** Estimators of bacterial diversity in rhizosphere soil of Leymus chinensis at different length of the dry intervals samples. Results from linear mixed model (LMM) are presented as F- and P value.

| Factors | Sum of sqrs | df | F | p |
| --- | --- | --- | --- | --- |
| amounts | 0.0207 | 4 | 0.478 | 0.751 |
| intervals | 0.0099 | 2 | 0.455 | 0.637 |
| Interaction | 0.0529 | 8 | 0.612 | 0.763 |

**Table S2.** Estimators of topological properties of the bacterial co-occurrence networks in different rhizosphere soil of *Leymus chinensis* at different length of the dry intervals.

|  |  | Nodes | Edges | Degrees | Clustering coefficient |
| --- | --- | --- | --- | --- | --- |
| Day 6 | CK | 2031.75±20.02 | 17111.25±245.79 | 16.84±0.22 | 0.49±0 |
|  | DP | 1989.25±69.82 | 16371±1580.06 | 16.43±1.05 | 0.49±0.01 |
|  | IP | 2039.5±38.91 | 17116.25±239.49 | 16.79±0.27 | 0.49±0 |
| Day 9 | CK | 2027.75±23.26 | 6410.75±104.58 | 6.32±0.04 | 0.27±0.01 |
|  | DP | 2032.75±24.53 | 6349.5±168.77 | 6.25±0.1 | 0.27±0.01 |
|  | IP | 2043.5±13.96 | 6511.25±146.85 | 6.37±0.1 | 0.28±0.01 |
| Day 12 | CK | 2059.25±25.45 | 4954.75±121.39 | 4.81±0.07 | 0.24±0.02 |
|  | DP | 2019.25±24.4 | 4651.25±173.9 | 4.61±0.12 | 0.23±0.01 |
|  | IP | 2052±14.31 | 4892.25±105.44 | 4.77±0.07 | 0.23±0.01 |
| Day 15 | CK | 2052.25±17.46 | 5786.5±125.12 | 5.64±0.12 | 0.38±0.02 |
|  | DP | 2033.75±23.51 | 5683.5±247.67 | 5.59±0.19 | 0.39±0.03 |
|  | IP | 2050±24.39 | 5705.5±251.02 | 5.57±0.19 | 0.38±0.02 |
| Day 18 | CK | 2042.25±47.03 | 7947.25±203.44 | 7.78±0.03 | 0.35±0 |
|  | DP | 2036±17.93 | 7982±86.81 | 7.84±0.06 | 0.34±0 |
|  | IP | 2017.25±30.08 | 7798±235.2 | 7.73±0.15 | 0.35±0 |
|  | Day | F=1.281 | F=2066.14*** | F=4461.095*** | F=630.202 *** |
|  | Precipitation | F=2.591 | F=2.915 | F=2.307 | F=0.127 |
|  | interaction | F=0.924 | F= 0.951 | F=0.870 | F= 0.750 |

Results from linear mixed model (LMM) are presented as F- and P value (* means p < 0.05, ** means p < 0.01 and *** means p < 0.001)., With the plot position serial number in the field as a random effect

**Table S3.** The keystone taxonomic composition in different rhizosphere soil of *Leymus chinensis* at different length of the dry intervals. See excel file.

**Table S4**. Results (F-values) of two-way ANOVAs for the effects of rainfall amount and dry interval on soil properties

|  |  | AP | SWC | NH4+-N | NO3−-N | P |
| --- | --- | --- | --- | --- | --- | --- |
| Day 6 | CK | 13.56±1.84 | 8.05±0.11b | 13.39±1.63 | 24.84±4.14a | 0.13±0.02 |
|  | DP | 16.58±1.84 | 5.15±0.06c | 14.09±2.86 | 35.63±6.98ab | 0.14±0.01 |
|  | IP | 12.86±1.92 | 9.73±0.01a | 11.81±2.82 | 21.67±3.22b | 0.12±0.01 |
| Day 9 | CK | 14.9±4.84 | 6.87±0.08b | 11.52±3.39 | 25.24±1.91ac | 0.12±0.01 |
|  | DP | 15.22±2.23 | 5.84±0.09c | 12.33±1.91 | 32.35±5.34a | 0.12±0.01 |
|  | IP | 13.01±3.91 | 8.93±0.19a | 10.53±2.9 | 17.23±3.37b | 0.11±0.01 |
| Day 12 | CK | 12.39±3.47 | 8.91±0.26b | 10.25±2.04 | 17.98±4.01a | 0.13±0.01 |
|  | DP | 12.31±1.62 | 7.72±0.14c | 12.27±1.1 | 23.94±3.91ab | 0.13±0.02 |
|  | IP | 10.32±2.11 | 10.63±0.24a | 9.34±2.14 | 14.93±2.9a | 0.12±0.02 |
| Day 15 | CK | 11.46±4.57 | 11.8±0.05b | 7.77±1.88 | 16.54±4.38a | 0.14±0.04 |
|  | DP | 12.41±1.28 | 8.12±0.09c | 8.5±1.74 | 22.6±3.42ab | 0.15±0.02 |
|  | IP | 10.07±1.98 | 14.72±0.09a | 7.36±1.53 | 12.53±4.53a | 0.12±0.03 |
| Day 18 | CK | 10.06±3.39 | 13.75±0.12b | 9.3±1.68 | 12.55±2.41a | 0.14±0.02 |
|  | DP | 11.06±2.84 | 11.33±0.29c | 10.22±1.58 | 17.17±2.89ab | 0.14±0.03 |
|  | IP | 10.5±2.9 | 16.32±0.15a | 8.37±2.84 | 9.57±3.47b | 0.12±0.01 |
|  | Day | F=4.505** | F=3983*** | F=9.642*** | F=25.57*** | F=1.753 |
|  | Precipitation | F=2.741 | F=4229*** | F=4.028* | F=40.09*** | F=3.897* |
|  | interaction | F=0.924 | F= 111*** | F=0.870 | F= 0.750 | F= 0.373 |

Results from linear mixed model (LMM) are presented as F- and P value (* means p < 0.05, ** means p < 0.01 and *** means p < 0.001)., With the plot position serial number in the field as a random effect


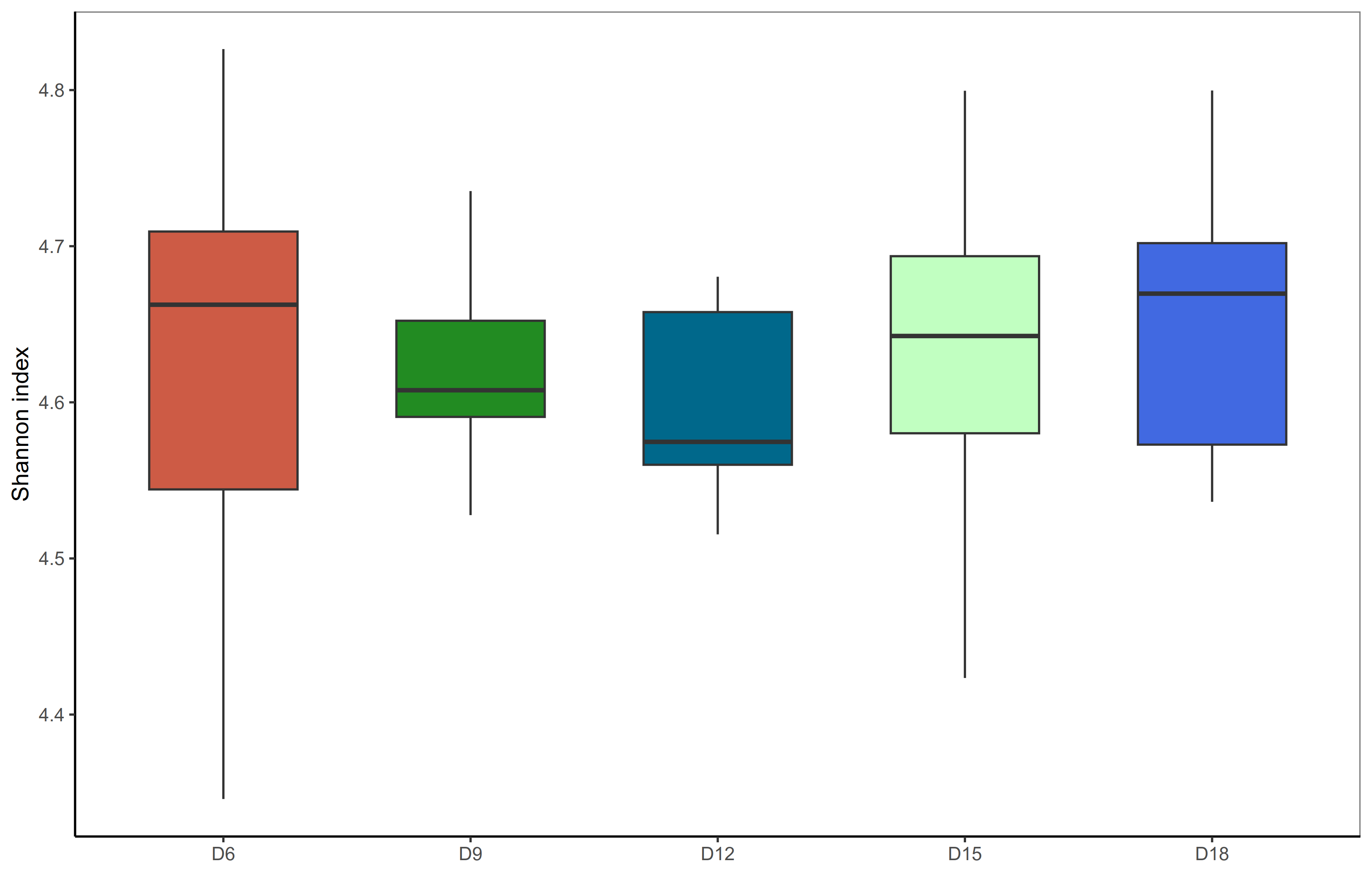


**Figure S1.** Alpha diversity of bacterial community in rhizosphere soil of *Leymus chinensis* at different length of the dry intervals. D6: 6 days dry intervals; D9: 9 days dry intervals; D12: 12 days dry intervals; D15: 15 days dry intervals; D18: 18 days dry intervals.

.


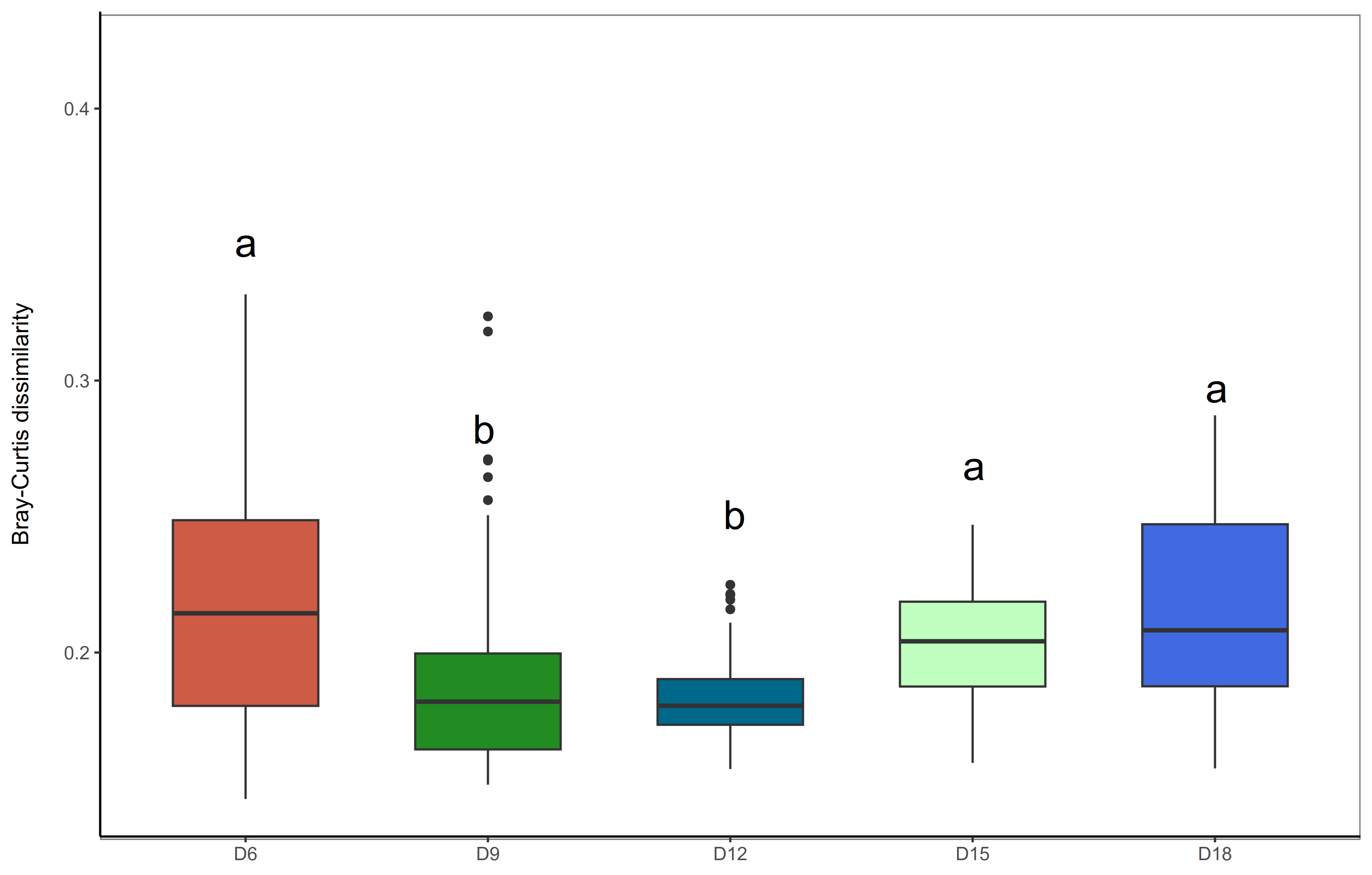


**Figure S2.** Beta diversity of bacterial community in rhizosphere soil of *Leymus chinensis* at different length of the dry intervals. D6: 6 days dry intervals; D9: 9 days dry intervals; D12: 12 days dry intervals; D15: 15 days dry intervals; D18: 18 days dry intervals.


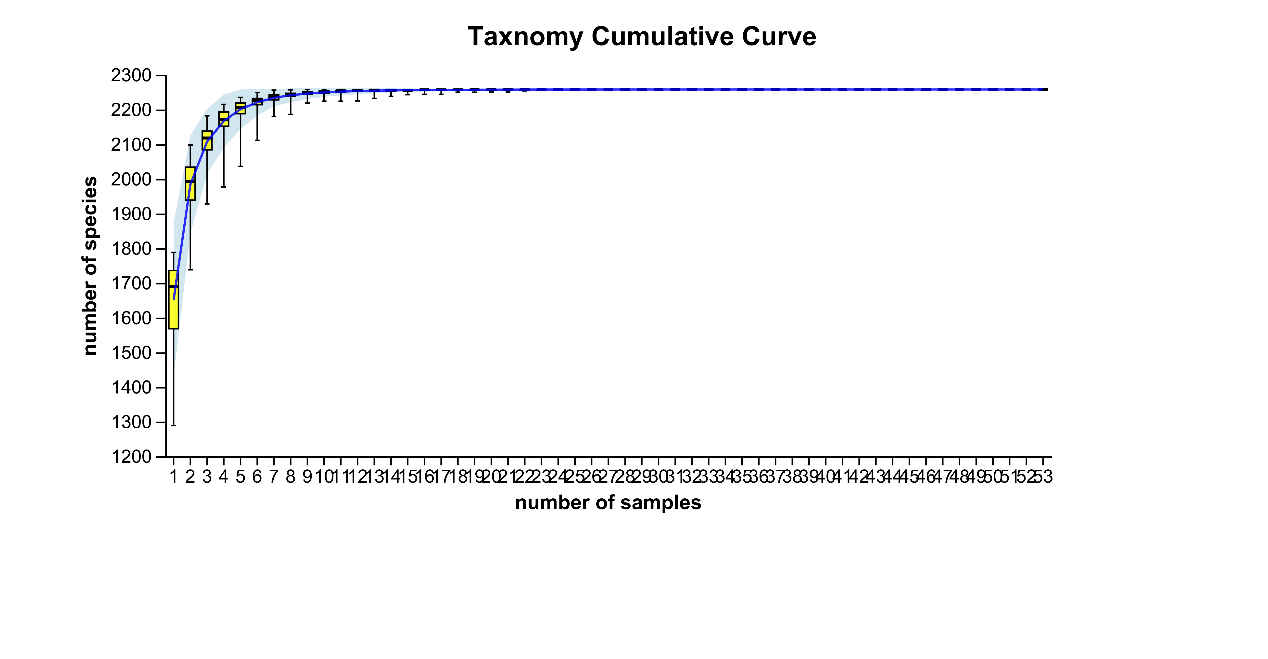


**Figure S3.** Taxa accumulation curves of bacteria in rhizosphere soil of *Leymus chinensis* at different length of the dry intervals.
